# Lower Zinc Bioavailability May Be Related to Higher Risk of Subclinical Atherosclerosis in Korean Adults

**DOI:** 10.1371/journal.pone.0080115

**Published:** 2013-11-06

**Authors:** Su Kyoung Jung, Mi-Kyung Kim, Young-Hoon Lee, Dong Hoon Shin, Min-Ho Shin, Byung-Yeol Chun, Bo Youl Choi

**Affiliations:** 1 Department of Preventive Medicine, College of Medicine, Hanyang University, Seoul, South Korea; 2 Institute for Health and Society, Hanyang University, Seoul, South Korea; 3 Department of Preventive Medicine & Institute of Wonkwang Medical Science, Wonkwang University College of Medicine, Iksan, South Korea; 4 Department of Preventive Medicine, School of Medicine Keimyung University, Daegu, South Korea; 5 Department of Preventive Medicine, Chonnam National University Medical School, Gwangju, South Korea; 6 Department of Preventive Medicine, School of Medicine, and Health Promotion Research Center, Kyungpook National University, Daegu, South Korea; William Harvey Research Institute, Barts and The London School of Medicine and Dentistry, Queen Mary University of London, United Kingdom

## Abstract

**Background:**

There is a proposed link between dietary zinc intake and atherosclerosis, but this relationship remains unclear. Phytate may contribute to this relationship by influencing zinc bioavailability.

**Objective:**

The aim of this study is to examine the relationship between zinc bioavailability and subclinical atherosclerosis in healthy Korean adults.

**Materials and Methods:**

The present cross-sectional analysis used baseline data from the Korean multi-Rural Communities Cohort Study (MRCohort), which is a part of The Korean Genome Epidemiology Study (KoGES). A total of 5,532 subjects (2,116 men and 3,416 women) aged 40 years and older were recruited from rural communities in South Korea between 2005 and 2010. Phytate:zinc molar ratio, estimated from a food-based food frequency questionnaire (FFQ) of 106 food items, was used to determine zinc bioavailability, and carotid intima media thickness (cIMT) and pulse wave velocity (PWV) were measured to calculate the subclinical atherosclerotic index.

**Results:**

We found that phytate:zinc molar ratio is positively related to cIMT in men. A higher phytate:zinc molar ratio was significantly related to an increased risk of atherosclerosis in men, defined as the 80^th^ percentile value of cIMT (5^th^ vs. 1^st^ quintile, OR = 2.11, 95% CI 1.42-3.15, *P* for trend = 0.0009), and especially in elderly men (5^th^ vs. 1^st^ quintile, OR = 2.58, 95% CI 1.52-4.37, *P* for trend = 0.0021). We found a positive relationship between phytate:zinc molar ratio and atherosclerosis risk among women aged 65 years or younger. Phytate:zinc molar ratio was not found to be related to PWV.

**Conclusions:**

Lower zinc bioavailability may be related to higher atherosclerosis risk.

## Introduction

Zinc is an essential micronutrient that plays catalytic, structural, and regulatory roles [1] and also has antioxidant and anti-inflammatory effects [2] in many organisms [3]. Zinc is closely related to many chronic diseases [1], such as cardiovascular diseases [4], cancers [5], autoimmune diseases [2], and liver diseases [6].

The bioavailability of nutrients is defined by the fraction of intake that can be absorbed into the blood system and used for physiologic function by the body. Various dietary factors can influence zinc bioavailability, but phytate is known to be the major dietary inhibitor [7]. A typical Korean diet consists of phytate-rich foods with low zinc content, such as grains, cereals, legumes, and vegetables [8]. Phytate itself may have beneficial effects in protecting against various cancers, heart-related diseases, diabetes mellitus, and renal stones [9]. However, because phytate may lead to mineral deficiency by forming insoluble substances with minerals like zinc, it should be considered in studies on zinc status [10]. Among several indicators to assess zinc status [11], phytate:zinc molar ratio may provide a useful index for dietary zinc bioavailability [12].

Subclinical atherosclerosis is generally measured by carotid intima media thickness (cIMT) and pulse wave velocity (PWV). However, these measurements reflect different aspects of atherosclerosis [13]. cIMT is widely used as a surrogate marker for atherosclerosis [14] and is regarded as a sensitive, reliable, convenient, and noninvasive method to assess the presence and extent of early atherosclerosis [14,15]. Among PWV measurements, the brachial-ankle PWV (baPWV) is now widely utilized as an indicator of aortic PWV [16,17].

There are few reports on the relationship between zinc intake and subclinical atherosclerosis [10,18], and the findings have been inconclusive. In cross-sectional studies, no relationship between dietary zinc and cIMT was found in a US population [19], but an inverse relationship was found in a Korean population [10]. Furthermore, zinc bioavailability as it relates to phytate intake was not considered in these studies.

We therefore evaluated the relationship between subclinical atherosclerosis and zinc bioavailability using the phytate:zinc molar ratio in a healthy adult population in rural areas of Korea.

## Subjects and Methods

### Study population

The Korean Multi-Rural Communities Cohort Study (MRCohort) was initiated to identify risk factors for cardiovascular disease as a part of The Korean Genome Epidemiology Study (KoGES). Between January 2005 and February 2010, we recruited 9,696 adults aged 40 years and over living in Yangpyeong, Namwon, and Goryeong. Yangpyeong is located 45 km east of Seoul, the capital of South Korea, and Namwon and Goryeong are located in southwestern and southeastern areas of South Korea, respectively. The majority of the subjects were farmers and housewives. Subjects who reported a physician-diagnosed heart disease (n=614), stroke (n=296), or cancer (n=204), or who were taking medication for hypertension (n=1,764), diabetes mellitus (n=328), or dyslipidemia (n=48), were excluded. We also excluded subjects who reported implausible dietary intakes (<500 or >4000 kcal/d or more than 10 missing food items, or missing value of cooked rice) (n=53), and those who did not have data on alcohol intake (n=7), blood pressure (n=12), anthropometric measurements (height, weight, waist circumference) (n=24), smoking status (n=9), education level (n=17), regular exercise (n=46), or cIMT or baPWV (n=742). Finally, 5532 subjects (2116 men, 3416 women) were included in the analysis. This study was conducted in accordance with the Declaration of Helsinki and all procedures involving human subjects were approved by the Institutional Review Board (IRB) of Hanyang University, Chonnam National University, and Keimyung University. Written informed consent was obtained from all subjects.

### General characteristics, anthropometrics, and biochemical variables

Standardized protocols that were developed for the questionnaire and each examination procedure, including measurements of height and weight, blood pressure, and blood sampling, were followed to overcome the limitations of a multi-center study. All interviewers and examiners were trained by the same personnel from the coordinating center.

To determine general characteristics, including information on demographics, education, smoking status, alcohol consumption, exercise, medical history, and menstrual and reproductive histories, a structured questionnaire was used by trained interviewers. The criterion for higher education was over 12 years of schooling, and regular exercise was defined as ≥ 3 times per week and ≥ 30 min per session. Smoking status was classified as current smoker, past smoker, or non-smoker. Study subjects were asked their average frequency of alcohol consumption and the average amount of alcohol consumed to estimate daily alcohol consumption. Total daily alcohol consumption was calculated from the sum of the amounts of all alcoholic beverages consumed, expressed in grams of alcohol per day (g/d). Height was measured with a standard height scale to the nearest 0.1 cm, and weight was measured with a metric weight scale to the nearest 0.01 kg in light clothing without shoes. Body mass index (BMI) was calculated as weight (kg)/ height (m^2^). Waist circumference was measured half way between the lowest rib margin and the iliac crest. We measured blood pressure in a seated position from the right arm by auscultation using a standard sphygmomanometer and cuff. Two consecutive measurements of blood pressure were taken after each subject had been sitting for at least 5 minutes. Systolic blood pressure (SBP) and diastolic blood pressure (DBP) were measured with a standard mercury sphygmomanometer using the first and fifth Korotkoff sounds, to the nearest 2 mmHg. If the two systolic or diastolic blood pressure readings were more than 5 mmHg apart, an additional measurement was performed, and the mean value of the last two measurements was used for the subsequent analyses. Blood samples were collected in the morning after at least eight hours of fasting and all biochemical markers were analyzed on the same day. Plasma total cholesterol, triglycerides, glucose, and high density lipoprotein (HDL) cholesterol concentrations were measured with an ADVIA1650 Automatic Analyzer (Siemens, New York, NY, USA). If plasma triglycerides were less than 400 mg/dl, low density lipoprotein (LDL) cholesterol was calculated as described by Friedewald et al. [20].

### Measurement of intima media thickness and pulse wave velocity

IMT was evaluated in the supine position using high-resolution B-mode ultrasound (SonoAce-9900; Medison Company Limited, Seoul, South Korea) equipped with a 7.5 MHz linear-array transducer. From the longitudinal view of the carotid bifurcation at a point 10mm proximal to the common carotid artery, the maximal value of the IMT in a region free of plaque was measured in both carotid arteries. End-diastolic images were captured and saved as files for offline analysis by a total of five sonographers from the three centers. A single trained reader at the reading center in the Department of Preventive Medicine at Chonnam National University analyzed the still images using Sigma Scan Pro 5.0 (SPSS Inc., Chicago, IL, USA). Between- and within-sonographer reliabilities were evaluated using 180 cases and 30 cases, respectively, and within-reader reliability was tested using 36 cases. The coefficient of variance (CV) between and within sonographers (9.4–9.8% and 4.7–4.9%, respectively), intraclass correlation coefficients between and within sonographers (r=0.85–0.88 and r=0.90–0.91, respectively), CV within readers (3.8–4.1%), and intraclass correlation coefficients within readers (r=0.92–0.94) all indicated that the IMT measurements taken at the three centers by five sonographers and one reader yielded highly reproducible values of cIMT.

Arterial stiffness was measured by baPWV defined as the distance between the right brachial site and each ankle site divided by the pulse wave transit time (PTT) from the ascending point of the right brachial pulse volume recording (PVR) to the ascending point of each ankle PVR (Colin VP-1000; Colin Co., Ltd., Komaki, Japan) [21]. Pearson’s correlation coefficient of reproducibility was r=0.961 and the inter-observer coefficient of variance was 5.4% (n=17) when a subsample was measured by a staff member [22]. In this study, the average value of the right and left baPWVs was calculated and used in the analyses.

### Dietary intake assessment

Food and nutrient intakes were estimated using a FFQ with 106 food items. Usual frequency of consumption of 106 food items during the past year and the average serving size consumed were assessed on the questionnaire. The FFQ consisted of nine frequency categories ranging from “never or rarely” to “3 times/d”, and three serving sizes were specified for each food item. For food items with limited seasonal availability, the participants were asked to mark whether they ate them for 3, 6, 9, or 12 months of the year. The validity and reproducibility of the FFQ have been examined in detail elsewhere [23]. Nutrient intake was calculated using a weighted frequency per day and serving size per unit for each food item. The seventh edition of the Korean Food Composition Table was used as the nutrient database [24].

### Phytate intake assessment and calculation of phytate:zinc molar ratio

Dietary phytate intake was estimated using phytate databases of commonly consumed foods in Korea [8] and the United States [25]. For foods with no information on phytate content [8,25], phytate values for different forms of the same food or for similar foods were substituted. For multi-ingredient dishes, phytate values were estimated by adding together values for the individual ingredients for the dish [26]. Phytate contents of sugar, coffee creamer, carbonated drinks, beverages, candy and chocolate, and jams/honey/butter/margarine were assumed to be zero, not only because they do not contain phytate, but also because the phytate intake from these sources would be small and would not considerably affect the relationship between zinc bioavailability and atherosclerosis.

Phytate:zinc molar ratios were determined from the molar intake of phytate (molecular weight, 660.1) and zinc (molecular weight, 65.4) using the formula phytate:zinc molar ratio = (mol of phytate intake)/(mol of zinc intake) [26]. 

### Statistical analysis

To avoid bias, nutrient intakes were adjusted for total energy intake by the residual method, which is based on the simple relationship between nutrient intake and total energy intake, in men and women separately [27]. Subjects were categorized into quintiles by daily phytate:zinc molar ratio. We performed a stratification analysis according to the age of the subjects. Subjects under 65 years of age were considered ‘the middle-aged’ and those over 65 years were considered ‘the elderly’.

We have described the general characteristics of the subjects using averages and standard deviations for continuous variables and prevalence for categorical variables. To assess potential confounders that could affect the relationship between phytate:zinc molar ratio and subclinical atherosclerosis, age-adjusted averages or prevalences were obtained by phytate:zinc molar ratio groups using the general linear model (GLM) and Cochran Mantel Haenszel analysis. Tukey's post-hoc comparison test was used to identify group differences at *P*< 0.05. The trend tests were conducted by treating the median value of each group as a continuous variable in the age-adjusted model. Variables showing significant linear trends across phytate:zinc molar ratio groups were included in the analysis as potential confounders. In each analysis, three different models were applied. The only variable that was adjusted in the first model was age. In the second model, variables that showed significant linear trends across quintiles of phytate:zinc molar ratio, except dietary variables, were adjusted (age, alcohol intake for men; age, alcohol intake, waist circumference, higher education, current smoker for women). Dietary variables were additionally adjusted in the third model (energy intake, protein intake, fat intake, carbohydrate intake, beta carotene intake, vitamin E intake, vitamin C intake for men; all dietary variables from the men’s analysis, except energy intake, for women).

cIMT was calculated as the mean IMT measurement of the right and left carotid arteries. To examine the means of cIMT and baPWV across phytate:zinc molar ratio groups, the GLM was used and Tukey’s post-hoc comparison test was used to identify group differences at *P*< 0.05. The trend tests were conducted by treating the median value of each group as a continuous variable in the multivariate-adjusted models.

Subclinical atherosclerosis was defined as ≥ 80^th^ upper percentile of cIMT (0.845 mm for men; 0.742 mm for women), which is similar to a study done by He et al. [28]. Unconditional logistic regression analysis was applied to obtain the odds ratios (ORs) and corresponding 95% confidence intervals (CIs) for subclinical atherosclerosis. The trend tests were conducted by treating the median value of each group as a continuous variable in the multivariate-adjusted models. Additionally, daily molar ratio of phytate:zinc was introduced as a continuous variable and expressed in increments of 5 ratios per day.

SAS software (version 9.1 SAS Institute Inc., Cary, NC, USA) was used for all statistical analyses and *P*-values < 0.05 were considered significant.

## Results

The general characteristics of the study subjects are shown in Table 1. The mean ages of the men and women were 61.3 y and 59.6 y, respectively. The proportions of subjects with higher education and who were current smokers and current drinkers were higher among men than among women. Men had a higher mean waist circumference, seated blood pressure, serum triglyceride level, and fasting blood glucose value than women. The mean baPWV and cIMT values were 1586.7 cm/sec and 0.736 mm for men, and 1491.6 cm/sec and 0.690 mm for women, respectively. The mean daily zinc intake for men and women was 6.7 mg and 5.7 mg, respectively. The average phytate:zinc molar ratio for men and women was 8.8 and 9.5, respectively.

**Table 1:** General characteristics of the study subjects.

Characteristics	Men	Women
n	2116	3416
Age (y)	61.3 ± 10.0	59.6 ± 10.0
Higher education (n, %)^†^	640 (30.0)	573 (16.6)
Regular exercise (n, %)‡	424 (20.0)	736 (21.5)
Current smoker (n, %)	720 (37.4)	105 (3.5)
Alcohol consumption		
Current drinker (n, %)	1424 (67.3)	1142 (33.4)
Alcohol intake (g/d)	29.3 ± 56.2	2.6 ± 12.8
Body mass index (kg/m^2^)	23.7 ± 2.9	24.2 ± 3.2
Waist circumference (cm)	84.7 ± 8.1	82.4 ± 9.0
Menopausal women (n, %)	-	2702 (79.4)
Seated blood pressure (mmHg)		
Systolic	124.8 ± 16.5	121.4 ± 17.8
Diastolic	80.0 ± 10.4	77.5 ± 10.2
Pulse rate (pulses/min)	65.7 ± 11.2	66.7 ± 10.1
Total cholesterol (mg/dL)	192.2 ± 34.9	204.4 ± 36.3
LDL cholesterol (mg/dL)	118.3 ± 31.4	131.4 ± 32.3
HDL cholesterol (mg/dL)	44.2 ± 11.7	45.7 ± 10.4
Triglyceride (mg/dL)	159.6 ± 119.1	138.8 ± 82.2
Fasting blood glucose (mg/dL)	100.4 ± 22.6	95.4 ± 14.9
baPWV (cm/sec)	1586.7 ± 341.6	1491.6 ± 335.8
cIMT (mm)	0.736 ± 0.149	0.690 ± 0.139
cIMT 80^th^ percentile (n, %)	424 (20.0)	684 (20.0)
Dietary intake		
Energy (kcal/d)	1742.4 ± 513.6	1520.3 ± 440.6
Protein (g/d)	50.9 ± 9.5	42.6 ± 8.1
Fat (g/d)	22.1 ± 8.9	15.5 ± 7.4
Carbohydrate (g/d)	309.2 ± 32.6	280.5 ± 28.3
β-carotene (µg/d)	1816.1 ± 1143.3	1602.8 ± 1090.6
Vitamin E (mg/d)	6.4 ± 2.1	5.6 ± 2.2
Vitamin C (mg/d)	81.4 ± 42.6	83.9 ± 48.1
Folate (µg/d)	170.0 ± 66.0	159.2 ± 66.4
Zinc (mg/d)	6.7 ± 1.6	5.7 ± 1.2
Phytate (mg/d)	575.5 ± 150.0	534.0 ± 130.1
Calcium (mg/d)	351.4 ± 162.4	331.9 ± 163.4
Phytate:zinc molar ratio	8.8 ± 2.4	9.5 ± 2.3
Phytate*calcium:zinc millimolar ratio	75.5. ± 39.3	76.1 ± 38.2

LDL, low density lipoprotein; HDL, high density lipoprotein; baPWV, brachial-ankle pulse wave velocity; cIMT, carotid intima media thickness

Values expressed as mean ± SD or number (%)

† ≥ High school graduates (12 years of education)

‡ ≥3 times/week and ≥30 min/session

Table 2 presents the potential confounders across phytate:zinc molar ratio groups. The median values of the quintiles of phytate:zinc molar ratio were 6.0, 7.5, 8.6, 9.9, and 11.8 in men, and 6.7, 8.2, 9.2, 10.6, and 12.5 in women. Age (years), daily alcohol intake (g/d), energy intake (kcal/d), carbohydrate intake (g/d), protein intake (g/d), fat intake (g/d), beta-carotene intake (µg/d), vitamin E intake (mg/d), and vitamin C intake (mg/d) for men, and age (years), waist circumference (cm), higher education (%), current smoker (%), daily alcohol intake (g/d), carbohydrate intake (g/d), protein intake (g/d), fat intake (g/d), beta-carotene intake (µg/d), vitamin E intake (mg/d), and vitamin C intake (mg/d) for women showed significant linear trends, and were adjusted for as potential confounders in the multivariate models.

**Table 2:** Age-adjusted characteristics of the study population according to phytate:zinc molar ratio group.

	Phytate:zinc molar ratio					
Characteristics	Q1	Q2	Q3	Q4	Q5	*P* for linear trend
***Men (n=2116)***						
n	423	423	424	423	423	
Median phytate:zinc molar ratio	6.0 (2.1-6.9)	7.5 (6.9-8.1)	8.6 (6.1-9.2)	9.9 (9.2-10.7)	11.8 (10.7-27.0)	
Median zinc intake (mg/d)	7.3 (3.5-21.6)	6.4 (3.9-10.5)	6.2 (3.5-9.8)	6.3 (3.0-9.0)	6.1 (3.4-8.6)	
Median phytate intake (mg/d)	435.1 (191.8-908.3)	474.3 (294.0-794.2)	535.0 (305.7-877.3)	627.4 (298.1-940.7)	738.7 (397.3-1539)	
Age (y)	60.0 ± 0.5^a^	61.5 ± 0.5^ab^	62.0 ± 0.5^b^	61.0 ± 0.5^ab^	62.5 ± 0.5^b^	0.0200
Body mass index (kg/m^2^)	23.9 ± 0.1	23.5 ± 0.1	23.6 ±0.1	23.8 ± 0.1	23.8 ± 0.1	0.1477
Waist circumference (cm)	85.6 ± 0.4^a^	84.0 ± 0.4^b^	83.9 ± 0.4^b^	84.7 ± 0.4^ab^	85.2 ± 0.4^ab^	0.9424
Higher education (%)†	30.1	31.8	27.8	33.3	27.3	0.6686
Current smoker (%)	39.5	36.7	37.7	34.4	35.0	0.0982
Current drinker (%)	69.9	69.4	65.6	66.2	64.7	0.0398
Alcohol intake (g/d)	37.3 ± 2.7^a^	29.3 ± 2.7^ab^	28.6 ± 2.7^ab^	26.8 ± 2.7^b^	24.5 ± 2.7^b^	0.0011
Regular exercise (%)‡	20.6	16.5	15.9	24.4	23.2	0.5833
Dietary intake*						
Energy (kcal/d)	1782.6 ± 23.3^a^	1676.1 ± 23.3^b^	1657.4 ± 23.3^b^	1792.8 ± 23.3^ac^	1803.4 ± 23.3^ac^	0.0266
Protein (g/d)	56.4 ± 0.4^a^	50.6 ± 0.4^b^	48.6 ± 0.4^c^	49.4 ± 0.4^bc^	49.3 ± 0.4^bc^	<.0001
Fat (g/d)	27.5 ± 0.4^a^	22.7 ± 0.4^b^	20.5 ± 0.4^c^	20.5 ± 0.4^c^	19.2 ± 0.4^c^	<.0001
Carbohydrate (g/d)	289.1 ± 1.5^a^	22.7 ± 0.4^b^	314.4 ± 1.5^c^	316.9 ± 1.5^c^	319.0 ± 1.5^c^	<.0001
β-carotene (µg/d)	2047.8 ± 55.3^a^	306.3 ± 1.5^b^	1717.4 ± 55.2^b^	1817.6 ± 55.3^b^	1659.8 ± 55.3^b^	<.0001
Vitamin E (mg/d)	7.0 ± 0.1^a^	6.3 ± 0.1^b^	6.2 ± 0.1^b^	6.4 ± 0.1^b^	6.1 ± 0.1^b^	<.0001
Vitamin C (mg/d)	90.3 ± 2.1^a^	81.8 ± 2.0^b^	77.6 ± 2.0^b^	82.6 ± 2.0^ab^	74.8 ± 2.0^b^	<.0001
Folate (µg/d)	181.5 ± 3.2^a^	167.5 ± 3.2^b^	159.7 ± 3.2^b^	169.3 ± 3.2^ab^	172.0 ± 3.2^ab^	0.1356
***Women (n=3416)***						
n	683	683	684	683	683	
Median phytate:zinc molar ratio	6.7 (1.9-7.7)	8.2 (7.7-8.7)	9.2 (8.7-9.8)	10.6 (9.8-11.4)	12.5 (11.4-21.1)	
Median zinc intake (mg/d)	6.1 (2.2-22.6)	5.4 (3.4-9.3)	5.5 (2.1-9.6)	5.4 (3.4-8.3)	5.4 (2.8-7.7)	
Median phytate intake (mg/d)	403.1 (135.6-897.9)	446.1 (278.2-733.9)	515.5 (188.0-945.5)	575.0 (335.6-871.7)	690.2 (357.0-1223)	
Age (y)	58.2 ± 0.4a	59.9 ± 0.4^b^	59.1 ± 0.4a^b^	59.2 ± 0.4a^b^	61.3 ± 0.4^c^	<.0001
Menopausal status (%)∥	79.2	78.9	79.3	79.4	79.8	0.5878
Body mass index (kg/m^2^)	24.2 ± 0.1	24.1 ± 0.1	24.1 ± 0.1	24.3 ± 0.1	24.4 ± 0.1	0.1288
Waist circumference (cm)	82.4 ± 0.3	82.0 ± 0.3	82.1 ± 0.3	82.5 ± 0.3	83.1 ± 0.3	0.0468
Higher education (%)†	18.9	16.9	17.0	16.1	13.6	0.0052
Current smoker (%)	4.5	4.2	3.7	2.1	2.6	0.0088
Current drinker (%)	41.4	33.1	33.7	30.7	28.2	<.0001
Alcohol intake (g/d)	3.3 ± 0.5	3.1 ± 0.5	2.5 ± 0.5	2.0 ± 0.5	1.9 ± 0.5	0.0153
Regular exercise (%)‡	22.6	21.4	20.1	21.3	21.9	0.8162
Dietary intake*						
Energy (kcal/d)	1524.2 ± 16.2a	1455.8 ± 16.2^b^	1548.2 ± 16.2a	1536.2 ± 16.2a	1536.2 ± 16.2a	0.0646
Protein (g/d)	46.5 ± 0.3a	41.6 ± 0.3^b^	41.6 ± 0.3^b^	41.9 ± 0.3^b^	41.9 ± 0.3^b^	<.0001
Fat (g/d)	20.1 ± 0.3a	15.0 ± 0.3^b^	16.7 ± 0.3^b^	14.1 ± 0.3^b^	14.1 ± 0.3^b^	<.0001
Carbohydrate (g/d)	265.8 ± 1.0a	281.6 ± 1.0^b^	283.5 ± 1.0^b^	284.1 ± 1.0^b^	284.1 ± 1.0^b^	<.0001
β-carotene (µg/d)	1740.5 ± 41.0a	1604.8 ± 41.0a^b^	1620.8 ± 40.9a^b^	1471.1 ± 41.1^b^	1471.1 ± 41.1^b^	<.0001
Vitamin E (mg/d)	6.1 ± 0.1a	5.6 ± 0.1^b^	5.5 ± 0.1^b^	5.3 ± 0.1^b^	5.3 ± 0.1^b^	<.0001
Vitamin C (mg/d)	92.2 ± 1.8^a^	82.7 ± 1.8^b^	83.1 ± 1.8^b^	77.8 ± 1.8^b^	77.8 ± 1.8^b^	<.0001
Folate (µg/d)	166.9 ± 2.^5a^	151.4 ± 2.^5b^	157.7 ± 2.^5ab^	160.1 ± 2.^5ab^	160.1 ± 2.5^ab^	0.1356

All results except each median value and age were adjusted for age, and all nutrient intakes are total energy-adjusted values.

Values are expressed as means ± SE or percent.

a,b, c Mean values with unlike superscript letters within a row were significantly different among the three groups by Tukey’s multiple comparison test.

*P* for linear trend was determined by the general linear model for continuous variables and by the Cochran-Mantel-Haenszel test for categorical variables.

∥The proportion among women subjects.

† ≥ High school graduates (12 years of education)

‡ ≥3 times/week and ≥30 min/session

The adjusted means of cIMT and baPWV by phytate:zinc molar ratio group are shown in Table 3. After adjusting for potential confounders such as age (years), daily alcohol intake (g/d), and dietary factors (energy intake, carbohydrate intake, protein intake, fat intake, beta-carotene intake, vitamin E intake, vitamin C intake), a significant positive relationship between phytate:zinc molar ratio and cIMT was found in men (*P* for trend=0.0080). There was no significant relationship found between baPWV and phytate:zinc molar ratio in men or women.

**Table 3:** Subclinical atherosclerosis index in the study subjects according to daily phytate:zinc molar ratio group.

	Phytate:zinc molar ratio						
	Q1	Q2	Q3	Q4	Q5	*P*-value*	*P* for linear trend
**Men (*n=2116*)**							
n	423	423	424	423	423		
Median phytate:zinc molar ratio	6.0	7.5	8.6	9.9	11.8		
Range of phytate:zinc molar ratio	(2.1-6.9)	(6.9-8.1)	(6.1-9.2)	(9.2-10.7)	(10.7-27.0)		
***Carotid intima media thickness (mm)***							
Adjusted cIMT mean 1	0.732 ± 0.006	0.726 ± 0.006	0.737 ± 0.006	0.738 ± 0.006	0.747 ± 0.006	0.2045	0.0388
Adjusted cIMT mean 2	0.733 ± 0.006	0.726 ± 0.006	0.737 ± 0.006	0.738 ± 0.006	0.746 ± 0.006	0.2507	0.0608
Adjusted cIMT mean 3	0.727 ± 0.007	0.726 ± 0.006	0.739 ± 0.006	0.738 ± 0.006	0.749 ± 0.006	0.0798	0.0080
***Pulse wave velocity (cm/sec)***							
Adjusted baPWV mean 1	1579.0 ± 15.0	1603.6 ± 15.0	1573.6 ± 15.0	1597.4 ± 15.0	1579.7 ± 15.0	0.5540	0.9162
Adjusted baPWV mean 2	1573.3 ± 14.9	1603.6 ± 14.9	1574.1 ± 14.8	1599.2 ± 14.9	1583.1 ± 14.9	0.4657	0.7509
Adjusted baPWV mean 3	1563.4 ± 15.7	1597.2 ± 14.9	1573.7 ± 14.9	1606.8 ± 14.9	1592.1 ± 15.1	0.2513	0.1984
**Women (*n=4316*)**							
n	683	683	684	683	683		
Median phytate:zinc molar ratio	6.7	8.2	9.2	10.6	12.5		
Range of phytate:zinc molar ratio	(1.9-7.7)	(7.7-8.7)	(8.7-9.8)	(9.8-11.4)	(11.4-21.1)		
***Carotid intima media thickness (mm)***							
Adjusted cIMT mean 1	0.688 ± 0.005	0.685 ± 0.005	0.685 ± 0.005	0.693 ± 0.005	0.698 ± 0.005	0.2193	0.0528
Adjusted cIMT mean 2	0.693 ± 0.007	0.690 ± 0.007	0.690 ± 0.007	0.698 ± 0.007	0.701 ± 0.007	0.3159	0.0838
Adjusted cIMT mean 3	0.690 ± 0.007	0.689 ± 0.007	0.690 ± 0.007	0.697 ± 0.007	0.700 ± 0.007	0.3090	0.0525
***Pulse wave velocity (cm/sec)***							
Adjusted baPWV mean 1	1489.8 ± 10.9	1497.2 ± 10.9	1490.4 ± 10.9	1475.5 ± 10.9	1505.2 ± 10.9	0.4031	0.6538
Adjusted baPWV mean 2	1474.1 ± 17.1	1482.2 ± 17.2	1475.2 ± 17.3	1459.9 ± 17.7	1490.0 ± 17.7	0.3843	0.6463
Adjusted baPWV mean 3	1463.1 ± 17.7	1482.2 ± 17.3	1475.3 ± 17.3	1461.7 ± 17.7	1491.5 ± 17.7	0.2636	0.2446

Values are expressed as means ± SE.

Q, quintile.*P values for differences across groups and P for linear trends were obtained using the general linear model (GLM).

Mean 1: Adjusted for age.

Mean 2: Adjusted for age, alcohol intake in men; adjusted for age, alcohol intake, waist circumference, higher education, smoking status in women.

Mean 3: Additionally adjusted for energy, protein, fat, carbohydrate, beta carotene, vitamin E, vitamin C in men; additionally adjusted for protein, fat, carbohydrate, beta carotene, vitamin E, vitamin C in women.

The relationships between phytate:zinc molar ratio and risk of atherosclerosis, which was defined as ≥ 80^th^ percentile value of cIMT (0.845 mm for men, 0.792mm for women), are shown in Table 4. In men, a positive relationship between phytate:zinc molar ratio and subclinical atherosclerosis risk was apparent in all multivariate adjusted models (Q5 vs. Q1, OR=2.11, 95% CI=1.42–3.15, *P* for trend=0.0009; ratio 5 unit/d OR=1.48, 95% CI=1.16–1.89, *P* value=0.0019 in third model). In elderly men (≥ 65 y), phytate:zinc molar ratio was positively related to subclinical atherosclerosis risk in all multivariate adjusted models (Q5 vs. Q1, OR=2.58, 95% CI=1.52–4.37, *P* for trend=0.0021; ratio 5 unit/d OR=1.53, 95% CI=1.12–2.11, *P* value=0.0080 in third model). No significant relationship was found in adult men <65y of age. There was no significant relationship between phytate:zinc molar ratio and subclinical atherosclerosis risk in women. However, in adult women <65 y of age, phytate:zinc molar ratio was positively related to subclinical atherosclerosis risk in the third model (Q5 vs. Q1, OR=1.62, 95% CI=1.03–2.55, *P* for trend=0.0392). 

**Table 4:** Risk of subclinical atherosclerosis index by daily phytate:zinc molar ratio group in multivariate models.

	Phytate:zinc molar ratio								
	Q1	Q2	Q3	Q4	Q5	*P* for trend*	Continuous, 5 ratio unit/d		*P*-value*
**Men (*Criterion:cIMT≥80*^*th*^* percentile (0.8445*))**									
***Total subjects (n=2116)***									
n	423	423	424	423	423				
Median (min-max)	6.0 (2.1-6.9)	7.5 (6.9-8.1)	8.6 (6.1-9.2)	9.9 (9.2-10.7)	11.8 (10.7-27.0)				
Subjects with cIMT≥80^th^ percentile (n, %)	59 (14.0)	83 (19.6)	93 (21.9)	88 (20.8)	101 (23.9)				
Multivariate-adjusted OR 1	1.00	1.40 (0.96-2.06)	1.56 (1.07-2.27)	1.57 (1.07-2.29)	1.79 (1.23-2.59)	0.0031	1.42 (1.13-1.80)		0.0029
Multivariate-adjusted OR 2	1.00	1.37 (0.93-2.01)	1.51 (1.03-2.20)	1.53 (1.05-2.24)	1.72 (1.19-2.50)	0.0057	1.39 (1.10-1.75)		0.0059
Multivariate-adjusted OR 3	1.00	1.66 (1.11-2.47)	1.91 (1.27-2.86)	1.86 (1.24-2.79)	2.11 (1.42-3.15)	0.0009	1.48 (1.16-1.89)		0.0019
***Subjects aged <65 years (n=1208)***									
n	269	239	228	246	226				
Median (min-max)	6.1 (2.8-6.9)	7.5 (6.9-8.1)	8.6 (8.1-9.1)	9.9 (9.2-10.7)	11.8 (10.7-20.7)				
Subjects with cIMT≥80^th^ percentile (n, %)	25 (9.3)	29 (12.1)	22 (9.7)	30 (12.2)	27 (12.0)				
Multivariate-adjusted OR 1	1.00	1.25 (0.70-2.21)	0.99 (0.54-1.82)	1.31 (0.74-2.32)	1.26 (0.70-2.25)	0.4296	1.22 (0.82-1.80)		0.3302
Multivariate-adjusted OR 2	1.00	1.23 (0.69-2.19)	0.98 (0.53-1.80)	1.29 (0.73-2.29)	1.22 (0.68-2.19)	0.4899	1.19 (0.80-1.76)		0.3923
Multivariate-adjusted OR 3	1.00	1.49 (0.82-2.70)	1.29 (0.68-2.45)	1.62 (0.89-2.97)	1.62 (0.87-3.03)	0.1385	1.40 (0.94-2.09)		0.1017
***Subjects aged* ≥65 years (*n=908*)**									
n	154	184	196	177	197				
Median (min-max)	5.9 (2.1-6.9)	7.4 (6.9-8.1)	8.6 (8.1-9.2)	9.9 (9.2-10.7)	11.8 (10.7-27.0)				
Subjects with cIMT≥80^th^ percentile (n, %)	34 (22.1)	54 (29.4)	71 (36.2)	58 (32.8)	74 (37.6)				
Multivariate-adjusted OR 1	1.00	1.56 (0.94-2.58)	2.06 (1.27-3.36)	1.80 (1.09-2.98)	2.28 (1.40-3.70)	0.0017	1.56 (1.16-2.09)		0.0030
Multivariate-adjusted OR 2	1.00	1.50 (0.91-2.49)	1.98 (1.21-3.22)	1.75 (1.06-2.90)	2.18 (1.34-3.55)	0.0030	1.52 (1.13-2.04)		0.0056
Multivariate-adjusted OR 3	1.00	1.85 (1.08-3.18)	2.47 (1.44-4.22)	2.12 (1.23-3.66)	2.58 (1.52-4.37)	0.0021	1.53 (1.12-2.11)		0.0080
**Women (*Criterion:cIMT≥80*^*th*^* percentile (0.792*))**									
***Total subjects (n=3416)***									
n	683	683	684	683	683				
Median (min-max)	6.7 (1.9-7.7)	8.2 (7.7-8.7)	9.2 (8.7-9.8)	10.6 (9.8-11.4)	12.5 (11.4-21.1)				
Subjects with cIMT≥80^th^ percentile (n, %)	121 (17.7)	133 (19.5)	126 (18.4)	137 (20.1)	167 (24.5)				
Multivariate-adjusted OR 1	1.00	0.94 (0.70-1.27)	0.95 (0.70-1.28)	1.08 (0.80-1.45)	1.21 (0.91-1.60)	0.0847	1.19 (0.99-1.44)		0.0709
Multivariate-adjusted OR 2	1.00	0.95 (0.71-1.28)	0.96 (0.71-1.30)	1.07 (0.80-1.44)	1.18 (0.89-1.57)	0.1338	1.17 (0.96-1.41)		0.1175
Multivariate-adjusted OR 3	1.00	0.99 (0.73-1.35)	1.00 (0.73-1.36)	1.11 (0.82-1.52)	1.21 (0.90-1.63)	0.1083	1.18 (0.97-1.44)		0.0977
***Subjects aged <65 years (n=2223)***									
n	482	428	450	445	418				
Median (min-max)	6.8 (2.3-7.7)	8.2 (7.7-8.7)	9.2 (8.7-9.8)	10.6 (9.8-11.4)	12.5 (11.4-21.1)				
Subjects with cIMT≥80^th^ percentile (n, %)	41 (8.5)	44 (10.3)	43 (9.6)	51 (11.5)	65 (15.6)				
Multivariate-adjusted OR 1	1.00	1.18 (0.75-1.88)	1.13 (0.71-1.80)	1.31 (0.83-2.05)	1.48 (0.96-2.27)	0.0667	1.26 (0.95-1.68)		0.1133
Multivariate-adjusted OR 2	1.00	1.21 (0.76-1.92)	1.16 (0.73-1.85)	1.32 (0.84-2.08)	1.46 (0.95-2.26)	0.0784	1.25 (0.94-1.66)		0.1325
Multivariate-adjusted OR 3	1.00	1.32 (0.82-2.13)	1.27 (0.78-2.04)	1.49 (0.93-2.39)	1.62 (1.03-2.55)	0.0392	1.30 (0.97-1.75)		0.0801
***Subjects aged* ≥65 years (*n=1193*)**									
n	201	255	234	238	265				
Median(min-max)	6.7 (1.9-7.7)	8.2 (7.7-8.7)	9.2 (8.7-9.8)	10.6 (9.8-11.4)	12.6 (11.4-18.1)				
Subjects with cIMT≥80^th^ percentile (n, %)	80 (39.8)	89 (34.9)	83 (35.5)	86 (36.1)	102 (38.5)				
Multivariate-adjusted OR 1	1.00	0.80 (0.54-1.18)	0.83 (0.56-1.23)	0.90 (0.61-1.33)	0.96 (0.66-1.40)	0.7963	1.08 (0.84-1.39)		0.5589
Multivariate-adjusted OR 2	1.00	0.80 (0.54-1.17)	0.84 (0.57-1.25)	0.91 (0.61-1.35)	0.97 (0.66-1.42)	0.7576	1.09 (0.84-1.40)		0.5200
Multivariate-adjusted OR 3	1.00	0.79 (0.52-1.19)	0.84 (0.56-1.28)	0.92 (0.60-1.39)	0.97 (0.65-1.45)	0.6677	1.11 (0.85-1.44)		0.4522

Values are expressed as odds ratios and 95 % confidence intervals.

Q, quintile.*P values for differences across groups and P for linear trends were obtained using the general linear model (GLM).

OR 1: Adjusted for age.

OR 2: Adjusted for age, alcohol intake in men; adjusted for age, alcohol intake, waist circumference, higher education, smoking status in women.

OR 3: Additionally adjusted for energy, protein, fat, carbohydrate, beta carotene, vitamin E, vitamin C in men; additionally adjusted for protein, fat, carbohydrate, beta carotene, vitamin E, vitamin C in women.

## Discussion

In this cross-sectional study, we evaluated the relationship between phytate:zinc molar ratio and atherosclerosis in healthy adults aged 40 years or more in Korea. In men, we found that phytate:zinc molar ratio was positively related to mean cIMT and a higher molar ratio was related to increased atherosclerosis risk defined by cIMT, particularly in the elderly subjects (aged ≥ 65 y). This positive relationship was also found in women aged < 65 y. However, there was no relationship between phytate:zinc molar ratio and baPWV for either men or women.

There are several studies that identified dietary zinc status using zinc intake, phytate:zinc molar ratio, or phytate*calcium:zinc millimolar ratio [8,26,29,30]. The average phytate:zinc molar ratio in the present study sample is lower (8.8 for men, 9.5 for women) than that in other sample populations of South Korea (9.5 for men, 10.5 for women, data from 841 (21-70 years old) adults living in Kuri city, South Korea [8]; 20.3, data from the 1995 National Nutrition Survey for South Korea [26]), in China sample (median value 11.1), and in United Kingdom sample (9.9-10.4 for men, 9.9-11.4 for women). In the present study, the average zinc intake for men and women (6.66 mg/d for men and 5.69 mg/d for women) was lower than the Korean estimated average requirements for dietary zinc [31], an adult sample from Kuri, Korea (9.5 mg/d for men and 7.5 mg/d for women) [8], an adult sample from China (8.5–13.3 mg/d) [29], and a European sample of elderly people (11.97–12.0 mg/d for men and 10.1–10.5 mg/d for women) [32]. With regard to average phytate intake, men and women had a lower intake (575.5 for men and 534.0 for women) than the adult sample from Kuri, Korea (839.4 mg/d for men and 752.3 mg/d for women) [8], the United Kingdom adult sample (923–1005 mg/d for men and 690–834 mg/d for women) [30], and the adult sample from China (823–1603 mg/d) [29].

Koreans typically consume a diet rich in phytate but low in zinc [8], which may lower zinc absorption. The major food sources contributing to dietary zinc intake were cereal grains (56%) followed by oysters (3%) and milk (3%) in a previous study [10], which is similar to our data (cereal 50%, oysters 6%, pork 3%, and milk 3% for men; cereal 55%, oysters 4%, and milk 4% for women). Although dietary zinc was mainly from cereal grains in the present study, oyster ingestion explained 31–46% of the total variation in dietary zinc intake. Dietary phytate food sources were also cereal grains (65%), followed by tofu (8%) and soybeans (3%) for men, and cereal grains (67%), tofu (8%), and rice cakes (3%) for women. For phytate intake, 23–28% of the total variation could be explained by cereal grain intake. The major food sources of zinc and phytate were common in the present study.

A few epidemiological studies have shown only inconclusive findings on the relationship between dietary zinc status and cIMT [10,19], as well as CVD risk [18]. Zinc intake was inversely associated with cIMT among Korean adults [10], but no association among a US population was observed [19]. In another prospective study targeting US adults, dietary zinc from red meat was positively associated with CVD risk [18]. Those studies did not consider zinc bioavailability. To the best of our knowledge, this is the first epidemiologic study to evaluate the relationship between zinc bioavailability and atherosclerotic measures such as cIMT and baPWV.

We found differences in the relationship between zinc bioavailability and cIMT based on sex. This result is in the line with a previous report that men commonly had a higher risk of heart disease than women [33]. A possible mechanism is still controversial, but sex hormones may affect cardiovascular metabolism differently in men and women [33]. Ovarian hormones, especially estrogen, may have a protective effect on cardiovascular events through various mechanisms [34]. However, this effect is not enough to explain the specific mechanism behind the sex differences found in the present study.

An age-stratified analysis was conducted in both men and women, and significant relationships between zinc bioavailability and cIMT were observed among elderly men and middle-aged women. No relation in the elderly women but the significantly positive relationship in middle-aged women is complicated to understand, although the relationship observed in elderly men is consistent with a previous report [35]. A previous review suggested that the sensitivity of zinc to phytate is likely to be significant only in diets predominantly based on unrefined cereals or pulses and high calcium intake. Therefore the sensitivity of zinc to phytate may not be significant in diet with low calcium content, even phytate-rich [36]. Although calcium intake was relatively low in the present study for all four groups (mean=295.6 mg/d for elderly women; 349.4 mg/d for elderly men; 351.4 mg/d for middle-aged women; 325.8 mg/d for middle-aged men), the lowest daily calcium intake for elderly women might partially explain the age differences.

The bioavailability of a nutrient can be defined as the effects of any process, physicochemical or physiological, that influences the fraction of an ingested trace element ultimately presented to tissues in forms that can be used to meet functional demands [36]. Bioavailability may be affected by the concentration of a nutrient, dietary factors, chemical form, supplements taken separately from meals, the nutrition and health status of the individual, excretory losses, and nutrient-nutrient interactions [37].

Zinc is an essential mineral for humans and whose absorption is inhibited by phytate. Zinc plays a potential role in the mechanism of cardiovascular disease, as it is known to interact with cardiovascular cells. There are two mechanisms to explain the role of zinc on cardioprotective action. First, increased oxidative stress in endothelial cells caused by oxidized LDL, long-chain fatty acids, or inflammatory cytokines leads to apoptosis [38]. Zinc decreases reactive oxygen species [21] as an NADPH oxidase inhibitor, and zinc is required for generating superoxide dismutase (SOD) and metallothionein (MT), which are known ROS inhibitor enzymes [39]. Second, zinc reduces inflammatory cytokines and adhesion molecules through inhibition of NF-kB activation by A20, zinc-finger protein [38,39]. However, dietary phytate inhibits zinc absorption [9], and has a negative effect on absorption and bioavailability of zinc [9]. Zn^2+^ easily combines with phytate in environments with pH 3–7, which is similar to conditions in the human intestine [40]. In the human gastrointestinal tract, which lacks phytate-degrading enzymes and microbial populations, dietary phytate forms an insoluble phytate-zinc complex and results in decreasing zinc absorption and bioavailability [9,41]. While there is no certain evidence of a direct relationship between dietary phytate and cardiovascular disease, an indirect relationship via inhibited zinc absorption may be important for cardioprotection [9].

In the present study, we found no relationship between baPWV and zinc bioavailability, in contrast to the positive relation found with cIMT. To our knowledge, there are no previous studies on the relationship of baPWV with zinc and zinc bioavailability to compare with the present study findings. However, as cIMT quantitatively measures arterial morphology in terms of intimal lesions and medial hypertrophy, and baPWV reflects arterial stiffening as a result of structural and functional changes of the vascular tree [13–16], these two metrics are looking at different aspects of atherosclerosis; baPWV and cIMT may both reflect arterial changes, but may have differential features [42]. In particular, the physiological effect of zinc on cardiovascular disease pathway is an inflammatory process, whereas arterial stiffness is a diffuse non-inflammatory fibrotic process [42]. Taken together, our findings suggest the possibility that cIMT, but not baPWV, may be mediated by an inflammatory response that involves zinc.

There are several limitations to consider when interpreting our findings. First, since the study is cross-sectional, we cannot draw causal conclusions concerning the relationship between phytate:zinc molar ratio and subclinical atherosclerosis risk. Second, as there is no national database for phytate, we used data from previously published literature [8,25]. Third, 385 of our study subjects (131 men, 254 women) were taking multivitamin supplements; however, most of them could not estimate zinc consumption from supplementation, either because they did not know the supplement brand name or due to a lack of data on the supplement nutrient content. Despite these limitations, the strength of the present study is that this is the first epidemiological study on zinc bioavailability and the risk of subclinical atherosclerosis. Another strength is to use two different non-invasive markers of CVD (cIMT and baPWV) which may reflect different aspects of subclinical atherosclerosis, because their combination is suggested to be an effective strategy to improve prediction of cardiovascular risk [43].

In conclusion, a higher intake of phytate relative to dietary zinc may be positively related to atherosclerosis risk. However, foods rich in phytate, such as whole grains and vegetables, may also be rich in antioxidants and anti-inflammatory substances. Therefore, we need to develop appropriate meal patterns that consider zinc bioavailability. Further prospective studies are needed to confirm this relationship.
